# Development and Validation of an HPLC Method for Simultaneous Assay of MCI and MI in Shampoos Containing Plant Extracts

**DOI:** 10.1155/2019/1851796

**Published:** 2019-11-20

**Authors:** Le Thi Huong Hoa, Vo Tran Ngoc Hung, Do Thu Trang, Thai Nguyen Hung Thu, Dinh Chi Le

**Affiliations:** ^1^National Institute of Drug Quality Control, Ministry of Health, Hanoi, Vietnam; ^2^Centre of Drug Quality Control of Quang Tri Province, Dong Ha, Vietnam; ^3^Department of Analytical Chemistry and Toxicology, Hanoi University of Pharmacy, Hanoi, Vietnam

## Abstract

A simple, easy-to-implement HPLC method was developed and validated for simultaneous determination of two isothiazolinone preservatives, methylchloroisothiazolinone (MCI) and methylisothiazolinone (MI), in hair care shampoo containing plant extracts. In this method, shampoo samples were first dissolved in isopropyl myristate and then MCI and MI were extracted from isopropyl myristate layer by a mixture of methanol and 0.02 M phosphate buffer solution pH 3.0 (30: 70, v/v) and analyzed on an analytical biphenyl column maintained at 25°C with a mixture of methanol and water (10: 90, v/v) in isocratic elution mode as mobile phase. Total flow rate of mobile phase was maintained at 1.0 mL per minute. The UV detection was performed at 274 nm. Injection volume was 50 *μ*l. The method was fully validated in terms of specificity, linearity, precision, accuracy, and robustness according to requirements of AOAC International and was proved as reliable and suitable for the intended application.

## 1. Introduction

The use of preservative is necessary for many types of cosmetics and toiletries because certain components in these products, such as plant extracts, can be favorable for the development of microorganism. Isothiazolinone-type biocides are effective preservatives with antimicrobial activity against a broad spectrum of fungi and bacteria [1]. Their effectiveness at low concentrations made these biocides a common choice for preservatives in cosmetic products [2]. The most widely used biocides of this group are methylchloroisothiazolinone (MCI) and methylisothiazolinone (MI), in which MI can be used alone or MCI and MI can be used together in 3 : 1 combined mixture. However, MCI and MI have been known to cause contact dermatitis [2–5]. Therefore, the use of the mixture MCI-MI was restricted in the European Union at 15 ppm (0.015 mg/g) [6] or recommended at 7.5 ppm for leave-on products and 15 ppm for rinse-off products [7]. The use of MI independently without the presence of MCI was claimed as safe at not more than 100 ppm in European Union [6]. However, the use of isotriazolinone biocides in cosmetic products has became more and more restricted in the last few years. In 2017, ASEAN countries, including Vietnam, only accepted MCI-MI (3 : 1) mixture and MI for use in rinse-off products at levels not more than 15 ppm [8]. In Vietnam, the consumption of cosmetics has been rapidly increased in recent years, with commercial products coming from many sources, including highly dubious ones. Therefore, it is necessary to have reliable and easy-to-implement analytical methods for controlling the actual levels of bioactive components with potential risk for human health in cosmetics in order to assure that regulation requirements are respected. In case of components with low concentration in cosmetics like MCI and MI, the method must be able to minimize the interference from sample matrix, which can be very complex, and to detect specifically the analytes with suitable limits of detection and quantification. The interference from product matrices can be very challenging, such as those of shampoos containing plant extracts. These products can contain the extracts of many plants, such as Panax ginseng, Ginkgo biloba, Fallopia multiflora, Gleditsia australis, Eleusine indica, Oroxylum indicum, Ageratum conyzoides, Morus alba, Agastache rugosa, Cymbopogon citratus, and Ocimum gratissimum, just to cite the most frequently declared ones on the shampoo labels (see Table 1 for some examples). These plant extracts can be used separately or combined, and sometimes a shampoo can contain extracts of 7 different plants in its composition. To extract MCI and MI from cosmetics and toiletries, different approaches have been employed, including solid-phase extraction [9, 10], matrix solid-phase dispersion [11], or direct dissolving from sample matrix with different solvents [12–15]. However, within the limit of our bibliographic research, we did not find any sample preparation process dealing with the matrix of shampoo containing plant extracts. The analysis of MCI and MI has been executed with gas chromatography coupled with mass spectrometric detection [16], but the majority of published works regarding the quantitative determination of MCI and MI in different types of samples have been carried out by using reverse-phase liquid chromatography on C18 column [10–15] or C30 column [9] with UV-Vis detection [9, 12] or mass spectrometric detection [10, 11, 13–15].

In this study, an HPLC method using phenyl column combined with sample treatment using liquid-liquid extraction was developed and validated for simultaneous assay of MCI and MI in shampoos containing plant extracts.

## 2. Materials and Methods

### 2.1. Instrumentation

Shimadzu LC-20AT HPLC system (Shimadzu, Kyoto, Japan) was used for method development and validation. This system was equipped with a pump (model LC-20AD), a degasser (model DGU-20A5), a photo diode array detector (model SPD-M20A), an autosampler (model SIL-20ACHT), and a control module (model CBM-20 Alite). The chromatographic separation was executed on a Apollo Phenyl column (250 × 4.6 mm, 5 *μ*m) of Hichrom (Lutterworth, Leicestershire, UK). Software LCsolution version 1.25 SP4 was used for data processing and evaluation.

### 2.2. Chemicals and Reagents

Reference substances of MI (purity: 98.2%) and MCI (purity: 96.0%) were purchased from Sigma Aldrich Singapore (Singapore). Methanol HPLC grade and other PA grade chemicals (potassium dihydrogenphosphate, orthophosphoric acid, and isopropyl myristate) were purchased from Merck Vietnam (Ho Chi Minh City, Vietnam). The 0.02 M phosphate buffer solution pH 3.0 was prepared by dissolving 2.72 g of potassium dihydrogenphosphate in 900 mL of water, adjusting the pH to 3.0 ± 0.1 by orthophosphoric acid if necessary and diluting with water to make 1000 mL.

For method development, 3 different shampoos containing plant extracts were used to optimize the conditions of sample preparation and chromatographic separation (see Table 1).

Blank sample for method validation containing the same components as those of Thai Duong 7 shampoo without MI and MCI and was kindly provided as a gift by Sao Thai Duong company (Vietnam).

### 2.3. Analytical Method

To obtain the suitable method, the conditions for sample preparation and chromatographic analysis were optimized in method development and optimization process, which is discussed in detail in 3.1. From the outcomes of this process, the final conditions of analytical method were fixed as in 2.3.1 and 2.3.2.

#### 2.3.1. Chromatographic Conditions

Mobile phase was mixture of methanol and water (10: 90, v: v). The flow rate of mobile phase was maintained at 1.0 mL/min. The analysis was carried out on an Shimadzu LC-20AT series HPLC system equipped with a photo diode array detector set at 274 nm for recording chromatograms. The chromatographic separation was conducted on a Apollo Phenyl column (250 × 4.6 mm, 5 *μ*m) maintained at 25°C. The injection volume was 50 *μ*l.

#### 2.3.2. Sample Preparation

*(1) Standard Solutions*. Stock standard solutions of MCI (1.0 mg/mL) and MI (1.0 mg/mL) were prepared by dissolving an accurately weighed quantity of corresponding reference standard in methanol. Working mix standard solutions were prepared by accurately diluting stock standard solutions to intended concentration using a mixture of methanol–0.02 M phosphate buffer solution pH 3.0 (30: 70, v: v). Standard solutions were filtered through 0.45 *μ*m membrane filter before being used for chromatographic analysis.

*(2) Sample Solution*. About 1.0 g of shampoo samples, accurately weighed into a 50-mL separation funnel, was dispersed in 10 mL of isopropyl myristate and extracted 2 times, each time by shaking for 10 minutes with 8 mL mixture of methanol–0.02 M phosphate buffer solution pH 3.0 (30: 70, v: v). The lower layer was then collected into a 20-mL volumetric flask and the mixture of methanol–0.02 M phosphate buffer solution pH 3.0 (30: 70, v: v) was added into the flask to make 20 mL. This solution was transferred into a centrifuge tube and left at 10°C in 30 minutes before centrifuging at 5000 rpm for 10 minutes at 10°C. A portion of supernatant was immediately filtered through 0.45 *μ*m filter membrane for chromatographic analysis.

*(3) Spiked Solutions*. For method development and validation, MI and MCI standards were added into blank sample at desired concentrations and then the spiked samples were treated with the process described in sample solution of 2.3.2.

*(4) Blank Solution*. About 1.0 g of blank sample was treated with the process described in sample solution of 2.3.2.

### 2.4. Method Validation

The conditions of final analytical method as being described in 2.3 were used for the method validation. The assessment of validation results was based on performance requirements of AOAC International [17] and those proposed in other published papers [18–23]. The method was validated in terms of specificity, linearity, sensitivity, accuracy, precision, robustness, and the stability of test solutions.

#### 2.4.1. Specificity

Specificity is the ability of the analytical method to distinguish between the analyte(s) and the other components in the sample matrix [18, 19]. In case of this HPLC method, it is assured by complete separation of MI and MCI from each other and from other peaks originated from sample matrix. Specificity evaluation was carried out by injecting separately 50 *μ*l of standard, sample, spiked sample, and blank into the chromatographic system.

#### 2.4.2. Linearity

To evaluate the linearity of the method, mixed standard solutions of MCI and MI were prepared by diluting stock standard solution with mixture of methanol–0.02 M phosphate buffer solution pH 3.0 (30: 70, v: v) to obtain different exact concentrations of MI (0.084, 0.135, 0.169, 0.203, and 0.253 *μ*g/mL) and MCI (0.255, 0.408, 0.510, 0.612, and 0.765 *μ*g/mL). Three injections from each concentration were analyzed under the same conditions. Linear regression analysis was used to evaluate the linearity of the calibration curve by using least square linear regression method, and the significance of linear regression was confirmed by one-way ANOVA test if *P* (Sig) < 0.05 [18].

#### 2.4.3. Sensitivity

The limit of detection (LOD) and limit of quantitation (LOQ) of MCI and MI were determined by analyzing different solutions of MCI and MI and measuring the signal-to-noise ratio for each analyte. The limit of detection (LOD) is the concentration giving a signal-to-noise ratio not less than 3 : 1, and the limit of quantitation (LOQ) is the concentration giving a signal-to-noise ratio not less than 10 : 1 with RSD of less than 10% with triplicate analysis [20, 21].

#### 2.4.4. Accuracy

The accuracy of the method was determined by recovery studies for MCI and MI from placebo matrix [17–19]. Exact amounts of reference substances of MCI and MI were mixed with blank matrix in such a way that the spiked samples, after preparation process, yielded solutions containing each analyte at three concentration levels within the linear range: at lowest concentration, at middle concentration, and at 80% of highest concentration of the calibration curve, i.e., about 0.084, 0.169, and 0.203 *μ*g/mL with MI and about 0.255, 0.510, and 0.612 *μ*g/mL with MCI. At each concentration level, nine samples were prepared and analyzed. The percentage recovery of added MCI and MI and the RSD were calculated for each replicate samples.

#### 2.4.5. Precision

The proposed method was validated in terms of system precision and method precision according to current guidelines and published papers [17, 18, 22].

The system precision was determined by six measurements of mixed standard solution containing about 0.169 *μ*g/mL of MI and 0.510 *μ*g/mL of MCI on the same day [18, 22]. The method precision includes repeatability and intermediate precision [17, 18, 22]. They were determined by estimating the dispersion of assay results obtained with recovery studies in 2.4.4 at each spiked level the same day and on two different days, respectively.

#### 2.4.6. Robustness

The robustness of the method was verified by investigating the effects caused by deliberate minor changes in experimental conditions to analyze the results [18, 22, 23]. In this study, following changes were applied:

- Flow rate: ±0.1 mL/min
- Percentage of methanol in mobile phase: ±1%

At each condition, a mixed standard solution containing about 0.169 *μ*g/mL of MI and 0.510 *μ*g/mL of MCI and three sample solutions of a shampoo product containing MI and MCI as preservatives were prepared and injected into chromatography system. The robustness of method was evaluated from the RSD of peak area for each analyte after three consecutive injections of standard solution and the RSD of the content of MCI and MI determined from sample solutions.

#### 2.4.7. Stability of Analytical Solution

The stability of analytical solutions was determined by analyzing the standard and sample solutions immediately after preparation and after 24 h in autosampler at 25°C. For each solution, three injections were executed at each time, and the stability of analytical solutions was evaluated from the variation of average peak area and RSD value of peak area among repeated injections.

### 2.5. Data Processing

SPSS software (version 16.0) of IBM SPSS Software (IBM, Armonk, NY, USA) was used for statistical analysis of analytical results.

## 3. Results and Discussion

### 3.1. Method Development and Optimization

**Figure 1:**
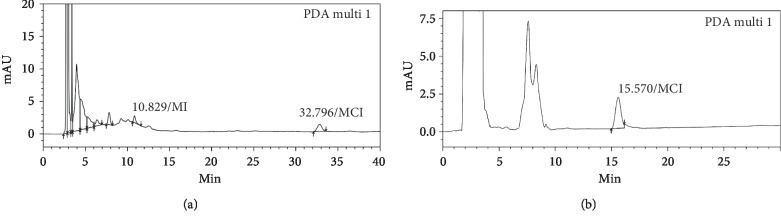
Some chromatograms obtained after analyzing placebo spiked with standard of MI and MCI during method development: (a) analysis on C18 column, sample preparation as per 2.4.2–Retention times were long for both analytes, especially MCI (more than 30 minutes); (b) analysis on biphenyl column with chromatographic conditions as per 2.3.1, sample was extracted directly with mixture methanol–0.02 M phosphate buffer solution pH 3.0 (30: 70, v: v) without previous dispersion in isopropyl myristate and cold centrifuging; peak of MI (retention time about 7.7 minutes) was not resolved.

**Figure 2:**
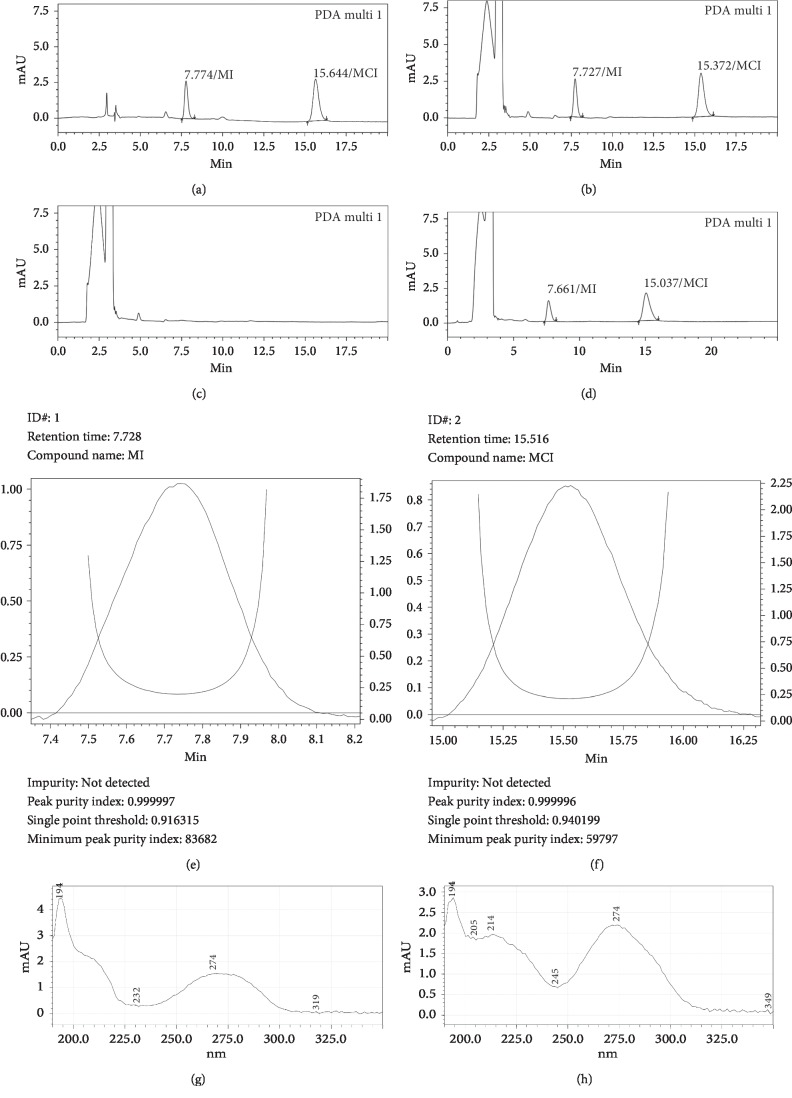
Chromatogram of mix standard solution (a), blank sample spiked with MI and MCI (b), blank sample (c), shampoo sample containing MI, MCI (d), peak purity (peak of MI (e) and peak of MCI (f)), and UV-Vis absorption spectra (spectrum of MI (g) and spectrum of MCI (h)) of analytes.

The matrix of shampoo with plant extracts was very complex due to the presence of many components coming from the various plants added into the product besides the normal composition of an usual shampoo. Direct dissolving of MI and MCI from this matrix has been carried out using methanol and mixtures of methanol–water, methanol–0.02 M phosphate buffer solution pH 3.0 at different percentages, and the obtained liquids were analyzed on C18 and phenyl stationary phases. With all above-mentioned solvent and solvent mixtures, analysis results revealed significant codissolving of other components from sample matrix. Due to low concentration of MI and MCI in shampoo and similar chromatographic behavior of many codissolved components, it was impossible to separate completely MI and MCI with C18 column. With phenyl column, the elution program permitting complete separation of MI and MCI from other matrix peaks was too long (more than 60 minutes per injection). However, HPLC analysis results showed that codissolving effect was less significant by using mixture methanol–0.02 M phosphate buffer solution pH 3.0 (30: 70, v: v) than by using methanol, methanol–water, and other ratio of methanol–0.02 M phosphate buffer solution pH 3.0 (preliminary results not shown). To reduce further the codissolving effect, the shampoo was first dissolved in isopropyl myristate before liquid-liquid extraction with mixture methanol–0.02 M phosphate buffer solution pH 3.0 (30: 70, v: v). By this process, MI and MCI were extracted effectively into the methanol–buffer phase due to their high polarity and the acidic pH of the methanol–buffer phase. The liquid obtained after liquid-liquid extraction step using conditions in 2.4.2 contained less coextracted components than the liquid obtained from direct dissolving (see Figure 1(b)). The cold centrifuge step after liquid-liquid extraction permitted further cleansing of the extracted liquid by reducing the solubility of certain coextracted components and eliminating them by precipitation, while the solubility of MI and MCI was not affected. This amelioration in sample preparation step permitted simplifying chromatographic conditions and faster analysis on phenyl column (only 18 minutes per injection, see Figure 2 for typical chromatograms and see 2.3 for chromatographic conditions). On C18 column, it was also possible to separate completely MI and MCI, but analysis time was longer than on phenyl column (more than 30 minutes per injection, (see Figure 1(a))). Therefore, liquid-liquid extraction combined with chromatographic analysis on biphenyl column has been selected for the final method. The UV-Vis absorption spectra of MI and MCI showed a maximal at wavelength about 274 nm; therefore, this wavelength was selected to record chromatograms (see Figures 2(g) and 2(h)).

### 3.2. Method Validation

#### 3.2.1. Specificity

Specificity was evaluated by comparing the chromatograms obtained after injecting separately 50 *μ*L of blank solution, spiked solution, standard solution, and sample solution prepared as per 2.4 into chromatographic system. The chromatogram results are shown in Figures 2(a)–2(d). In selected chromatographic conditions, MI and MCI each were eluted in one completely resolved peak. The peak of MI was eluted before the peak of MCI. It can be observed from the peak purity analysis (Figures 2(e) and 2(f)) that there are no coeluting peaks at the retention times of MCI and MI to interfere with peaks of analytes. This result indicated that the peak of the analyte was pure and this confirmed the specificity of the method.

#### 3.2.2. Linearity and Range

**Figure 3:**
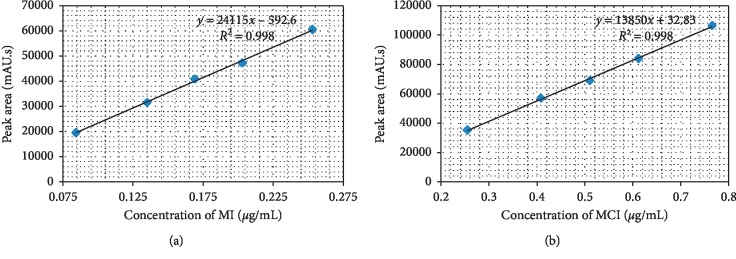
Calibration curves of MCI (a) and MI (b).

Analytical method linearity is defined as the ability of the method to obtain test results that are directly proportional to the analyte concentration, within a specific range. The mean peak area obtained from the chromatograms was plotted against corresponding concentrations to obtain the calibration graph. The results of linearity study (Figure 3) gave linear relationship over the concentration range of 0.084–0.253 *μ*g/mL for MI and of 0.255–0.765 *μ*g/mL for MCI. From the regression analysis, the linear equation was obtained: *y* = 241158*x* − 592.6 for MI and *y* = 138508*x* + 32.83 for MCI, and the coefficient of determination R-square was 0.998 for both analytes, indicating a linear relationship between the concentration of analyte and area under the peak. ANOVA analysis for both analytes (as shown in (Tables 2 and 3)). also proved that the regression model statistically significantly predicts the outcome variable (*P* < 0.05) [18].

#### 3.2.3. Limit of Detection (LOD) and Limit of Quantification (LOQ)

The limit of detection (LOD) is the lowest amount of analyte in a sample that can be detected, but not necessarily quantitated, while the limit of quantification (LOQ) is the lowest amount of analyte in a sample that can be quantitatively determined with suitable precision [18, 20, 21]. For MI, the concentration of injected solution at LOD and LOQ was 0.030 *μ*g/mL and 0.084 *μ*g/mL, equivalent to the content of MI in shampoo of 0.60 ppm and 1.68 ppm, respectively. For MCI, the concentration of injected solution at LOD and LOQ was 0.090 mg/mL and 0.255 mg/mL, equivalent to the content of MCI in shampoo of 1.80 ppm and 5.10 ppm, respectively. According to the current legal requirements in Vietnam [8], with the limit of mixture MCI-MI (3: 1, w: w) being set at 15 ppm, the acceptable content of MI and MCI in rinse-off cosmetics must be not more than 3.75 ppm and 11.25 ppm, respectively. With LOQ of both MI and MCI lower than the current legal acceptable limits, this method has suitable sensitivity for control of MI and MCI in shampoo for regulatory purposes.

#### 3.2.4. Accuracy

The accuracy of an analytical method expresses the closeness of results obtained by that method to the true value. In this study, the recovery rate fell within the range from 86.5% to 101.8% for the two analytes. At each level of concentration where recovery study was done, the RSD values varied from 2.5% to 5.2%, as shown in Table 4. These results were within the accepted limit for recovery each concentration level according to AOAC International [17].

#### 3.2.5. Precision

The precision of the method is defined as “the closeness of agreement between a series of measurements obtained from multiple sampling of the same homogeneous sample under the prescribed conditions,” [20] and it is normally expressed as the relative standard deviation.

In terms of system precision, the RSD of retention time, peak area, and the performance of chromatographic system, represented by the number of theoretical plate and tailing factor, were all less than 2.0%, and the number of theoretical plate was higher than 1000 for all analyte peaks, as shown in Table 5. In terms of method precision, the RSD of assay results for MCI and MI in the evaluation of repeatability and intermediate precision were all within the accepted limit for precision each concentration level according to AOAC International [17], as shown in Table 4. Therefore, the results of both system and method precision showed that the method is precise within the acceptable limits for intended application.

#### 3.2.6. Robustness

The analytical method robustness was tested by evaluating the influence of minor modifications in HPLC conditions on system suitability parameters of the proposed method, as mentioned in Section 2.4.6.

In the robustness test, a minor change of method conditions, such as the composition and flow rate of the mobile phase, caused variations in analytical results within acceptable limit, i.e., RSD less than 2.0% (Table 6). These results demonstrated that the method was robust in case of minor variations in experimental conditions. In all modifications, good separation was achieved between peak of MI and peak of MCI, as well as between peaks of these substances and other peaks on chromatograms, and the RSD values of peak area were obtained from repeated injections of standard solution. The RSD values of MI content and MCI content determined from Thai Duong 7 shampoo (Table 1) containing approximately 3 ppm of MI and 9 ppm of MCI were all less than 2.0% and lower than limit proposed by AOAC International for this range of concentration [17]. Furthermore, the minor changes applied in this robustness test produced no significant difference in content of MCI and MI found in sample, as one-way ANOVA analysis found *F* < *F*_inscrit_ for both analytes (as shown in Table 7).

#### 3.2.7. Solution Stability

The percentage of recovery was within the range of 98.0% to 102.0% and RSD was not more than 2.0%, indicating a good stability of the sample and standard solutions for 24 hr at 25°C in autosampler, as shown in Table 8. These results proved that both analytes were stable in sample and standard solutions prepared as described in 2.4, and the preparation procedure for sample and standard solution was suitable for intended application of the method.

## 4. Conclusion

In this study, a simple, accurate, precise, and robust HPLC method has been developed for simultaneous assay of MCI and MI in shampoos containing plant extracts. In the extent of our literature research, this was the first method using chromatographic separation on phenyl column and liquid-liquid extraction for analysis of MI, MCI in cosmetics. The method was validated according to the requirements of AOAC International on analytical method performance, proved to be suitable for the intended application, and able to provide accurate and precise quantitative results under minor variation of chromatographic conditions.

## Figures and Tables

**Table 1:** Information about shampoos containing plant extracts used for method development and validation.

Name of product	Manufacturer	Plant extracts (as declared on the label)
Thai Duong 7 shampoo	Sao Thai Duong company (Vietnam)	Gleditsia australis, ocimum gratissimum, morus alba, eleusine indica, ageratum conyzoides, oroxylum indicum, curcuma longa, andrographis paniculata

Clear with plant extract	Unilever Vietnam (Vietnam)	Panax ginseng, gleditsia australis, mentha arvensis, juniperus communis, camellia sinensis, cinnamomum verum, centella asiatica, lonicera japonica, eclipta alba

Hasuo herbal hair Care shampoo	Organia (South Korea)	Fallopia multiflora

**Table 2:** Results of ANOVA analysis for calibration curve of MCI.

Model		Sum of squares	Df	Mean square	*F*	Sig.
1	Regression	2.894*E*9	1	2.894E9	2.357*E*3	0.000^a^
	Residual	3684192.179	3	1228064.060		
	Total	2.898*E*9	4			

^a^Predictors: (constant), MCI_concentration. ^b^Dependent variable: MCI_peak_area.

**Table 3:** Results of ANOVA analysis for calibration curve of MI.

Model		Sum of squares	Df	Mean square	*F*	Sig.
1	Regression	9.650*E*8	1	9.650*E*8	1.704*E*3	0.000^a^
	Residual	1699090.618	3	566363.539		
	Total	9.667*E*8	4			

^a^Predictors: (constant), MI_concentration. ^b^Dependent variable: MI_peak_area.

**Table 4:** Results of accuracy and precision.

Replicate number (^*∗*^)	MI				MCI			
	Spiked amount of standard (*μ*g)	Peak area	Recovery (%)	Mean recovery (MR), RSD (%)	Spiked amount of standard (*μ*g)	Peak area	Recovery (%)	Mean recovery (MR), RSD (%)
*First spiked level (approximately the lowest concentration of calibration curve)*								
1	1.74	18343	90.3	Repeatability (1–6): MR: 92.6% RSD: 3.6% Intermediate precision (1–9): MR: 91.9% RSD: 3.7%	5.22	33042	91.3	Repeatability (1–6): MR: 92.2% RSD: 5.2% Intermediate precision (1–9): MR: 92.0% RSD: 4.9%
2	1.74	17957	88.4		5.22	34758	96.1	
3	1.74	18834	92.6		5.22	31895	88.1	
4	1.74	19762	97.0		5.22	32630	90.2	
5	1.74	18556	91.3		5.22	36114	99.8	
6	1.74	19504	95.8		5.22	31775	87.8	
7	1.74	17549	86.5		5.22	32675	90.3	
8	1.74	18631	91.6		5.22	35117	97.1	
9	1.74	19125	94.0		5.22	31626	87.4	

*Second spiked level (approximately the middle of concentration range of calibration curve)*								
1	3.40	40871	101.1	Repeatability (1–6): MR: 98.1% RSD: 3.2% Intermediate precision (1–9): MR: 97.1% RSD: 3.5%	10.16	67223	95.5	Repeatability (1–6): MR: 94.6% RSD: 2.7% Intermediate precision (1–9): MR: 94.0% RSD: 2.6%
2	3.40	39065	96.7		10.16	65024	92.4	
3	3.40	41127	101.8		10.16	66309	94.2	
4	3.40	40138	99.4		10.16	64117	91.1	
5	3.40	38722	95.9		10.16	67854	96.4	
6	3.40	37892	93.9		10.16	68973	98.0	
7	3.40	38205	94.6		10.16	65459	93.0	
8	3.40	37051	91.8		10.16	66715	94.8	
9	3.40	39726	98.3		10.16	63814	90.6	

*Third spiked level (approximately 80% of highest concentration of calibration curve)*								
1	4.12	44788	91.3	Repeatability (1–6): MR: 93.3% RSD: 2.5% Intermediate precision (1–9): MR: 93.6% RSD: 2.6%	12.20	81056	95.9	Repeatability (1–6): MR: 94.7% RSD: 3.4% Intermediate precision (1–9): MR: 94.4% RSD: 2.8%
2	4.12	46205	94.2		12.20	79682	94.3	
3	4.12	47631	97.1		12.20	83127	98.3	
4	4.12	45176	92.1		12.20	76531	90.5	
5	4.12	44507	90.8		12.20	82415	97.5	
6	4.12	46348	94.5		12.20	77214	91.3	
7	4.12	44501	90.8		12.20	78462	92.8	
8	4.12	47638	97.1		12.20	80574	95.3	
9	4.12	46152	94.1		12.20	79263	93.8	

^∗^ *Note*. For each spiked level, replicate analysis from No. 1 to No. 6 was done in the same day and used to validate the repeatability of the method, replicate analysis No. 7 to No. 9 was done in another day, and all replicate analysis from No. 1 to No. 9 was used together to validate the intermediate precision of the method.

**Table 5:** Results of system precision.

No. of injection	Retention time (minutes)	Peak area (mAu.s)	Asymmetry of peak	Number of theoretical plates	Resolution with nearest peak
*MI*					
1	7.774	41045	1.4	3196	2.7
2	7.703	40758	1.4	3138	2.7
3	7.721	42103	1.4	3153	2.7
4	7.684	40356	1.4	3123	2.7
5	7.625	41573	1.4	3075	2.7
6	7.672	40952	1.4	3113	2.7
Average	7.696	41131	1.4	3133	
RSD (%)	0.7	1.5	0.1	1.3	

*MCI*					
1	15.644	70761	1.1	4834	7.1
2	15.582	72623	1.1	4796	7.1
3	15.671	71658	1.1	4851	7.1
4	15.516	70216	1.1	4755	7.1
5	15.683	70458	1.1	4858	7.1
6	15.547	72054	1.1	4774	7.1
Average	15.607	71295	1.1	4812	
RSD (%)	0.4	1.3	0.1	0.9	

**Table 6:** Results of robustness.

Variation	Specific condition	MI		MCI	
		RSD (%) for peak area	RSD (%) for content in shampoo	RSD (%) for peak area	RSD (%) for content in shampoo
Flow rate (mL/min)	0.9	0.4	0.4	0.4	0.3
	1.0 (normal)	0.6	0.4	0.4	0.4
	1.1	0.6	0.5	0.6	0.3

Percentage of methanol in mobile phase (%)	29	0.8	0.6	0.4	0.6
	30 (normal)	0.6	0.5	0.4	0.3
	31	0.5	0.4	0.5	0.3

**Table 7:** Results of ANOVA analysis for content of MI and MCI.

		Sum of squares	df	Mean square	*F*	Sig.
content_MI	Between groups	0.001	5	0.000	1.126	0.398
	Within groups	0.002	12	0.000		
	Total	0.003	17			

content_MCI	Between groups	0.010	5	0.002	1.560	0.244
	Within groups	0.015	12	0.001		
	Total	0.024	17			

**Table 8:** Results of stability studies.

Studies	Average retention time (minutes)	Average peak area (mAu.s)	Average asymmetry of peak	Average number of theoretical plate	RSD (%) of peak area	Recovery (%)
MI						
(i) Standard solution						
Initial	7.695	41236	1.4	3131	0.3	—
After 24 h	7.703	40697	1.4	3027	0.4	98.7
(ii) Sample solution						
Initial	7.711	38263	1.4	3142	0.2	—
After 24 h	7.692	37851	1.4	3129	0.4	98.9

MCI						
(i) Standard solution						
Initial	15.638	71554	1.1	4940	0.2	—
After 24 h	15.674	70981	1.1	4852	0.3	99.2
(ii) Sample solution						
Initial	15.592	67083	1.1	4910	0.1	—
After 24 h	15.613	66348	1.1	4815	0.3	98.9

## Data Availability

The data used to support the findings of this study are available from the corresponding author upon request.
